# Lagoon, Anaerobic Digestion, and Composting of Animal Manure Treatments Impact on Tetracycline Resistance Genes

**DOI:** 10.3390/antibiotics11030391

**Published:** 2022-03-15

**Authors:** Getahun E. Agga, Melanie Couch, Rohan R. Parekh, Faranak Mahmoudi, Keerthi Appala, John Kasumba, John H. Loughrin, Eric D. Conte

**Affiliations:** 1 Food Animal Environmental Systems Research Unit, Agricultural Research Service, USDA, Bowling Green, KY 42101, USA; rohan.parekh@usda.gov (R.R.P.); john.loughrin@usda.gov (J.H.L.); 2 Department of Chemistry, Western Kentucky University, 1906 College Heights Blvd., Bowling Green, KY 42101, USA; melanie.couch@nscc.edu (M.C.); faranak.mahmoudi@wku.edu (F.M.); ka79422@uga.edu (K.A.); john.kasumba@regeneron.com (J.K.); eric.conte@wku.edu (E.D.C.)

**Keywords:** tetracycline resistance genes, lagoons, anaerobic digestion, composting, animal manure, antimicrobial stewardship, One Health

## Abstract

Increased demand for animal protein is met by increased food animal production resulting in large quantities of manure. Animal producers, therefore, need sustainable agricultural practices to protect environmental health. Large quantities of antimicrobials are used in commercial food animal production. Consequently, antimicrobial-resistant bacteria and the resistance genes emerge and are excreted through feces. Manure management is essential for the safe disposal of animal waste. Lagoons, with or without covers, and anaerobic digesters, with the primary purpose of methane production, and composting, with the primary purpose of producing organic fertilizer, are widely used methods of manure treatment. We reviewed manure management practices and their impact on tetracycline resistance genes. Lagoons are maintained at ambient temperatures; especially uncovered lagoons are the least effective in removing tetracycline resistance genes. However, some modifications can improve the performance of lagoons: sequential use of uncovered lagoons and the use of covered lagoons resulted in a one-log reduction, while post-treatments such as biofiltration following covered lagoon treatment resulted in 3.4 log reduction. Mesophilic digestion of animal manure did not have any significant effect; only a 0.7 log reduction in *tet*(A) was observed in one study. While thermophilic anaerobic digesters are effective, if properly operated, they are expensive for animal producers. Aerobic thermophilic composting is a promising technology if optimized with its economic benefits. Composting of raw animal manure can result in up to a 2.5 log reduction, and postdigestion composting can reduce tetracycline resistance gene concentration by >80%. In general, manure management was not designed to mitigate antimicrobial resistance; future research is needed to optimize the economic benefits of biogas or organic fertilizer on the one hand and for the mitigation of foodborne pathogens and antimicrobial resistance on the other.

## 1. Introduction

With the increasing human population, the global demand for animal protein is expected to rise [1]. This has led to increased food animal production with simultaneous production of high quantities of animal manure, resulting in environmental concerns putting enormous pressure on food animal producers for economically and environmentally sustainable solutions [2]. Modern commercial food animal production has become more intensified; consequently, large amounts of antibiotics are used to maintain animal health and productivity [3,4,5]. Global antimicrobial use in food animal production is increasing to support the growing number of animals raised for food production [6], and it is expected to rise by 11.5% in 2030 [3]. In food animal production, antimicrobials are used for therapeutic (to treat clinical infections), prophylactic (i.e., to prevent infections), or growth promotion purposes [7].

The use of antimicrobials is undoubtedly the most important factor for the increased occurrence of antimicrobial resistance [4]. Within the animal body, antimicrobials directly exert selection pressure giving a competitive advantage for the resistant strains, or indirectly through horizontal gene transfer if the resistance genes are carried on mobile genetic elements such as plasmids and transposons [7]. It is estimated that 75–90% of antibiotics administered to food animals are excreted, largely unmetabolized or as transformation products, into the environment through feces and urine that together constitute manure [8,9]. Excreted antibiotics and their transformation products cause in situ selection pressure in the native microbiota in the environment with subsequent development and spread of antibiotic-resistant bacteria [10,11,12]. Furthermore, preformed antibiotic-resistant bacteria and antimicrobial resistance genes (ARGs) can be excreted through animal manure [13].

Manure-borne antimicrobial resistance (AMR) determinants (i.e., resistant bacteria, resistance genes, and mobile genetic elements) can contaminate soil, water, and air (i) directly through runoffs from animal production facilities, (ii) through animal manure land application for crop production, (iii) through irrigation water from manure storage ponds and manure treatment facilities, and (iv) via airborne particulate matter [11,14,15]. From there, AMR determinants can spread from animals to humans through (i) food crops grown on manure amended soil, (ii) contamination of recreational, irrigation, and drinking water sources, or (iii) air pollution around animal production facilities and aerosolization during manure application. Animal manure management is required to dispose of the waste from animal production facilities; it is also a critical control point necessary for reducing the spread of antibiotic-resistant bacteria into the environment and beyond [4].

To mitigate the rising concern of AMR, the use of antibiotics for growth promotion purposes was banned in the European Union in 2006 [16]. In the United States, through the implementation of the Guidance for Industry #213 [17] by the Food and Drug Administration (FDA) as of 3 January 2017, the use of medically important antibiotics as defined in Guidance for industry #152 [18] for growth promotion purposes has been eliminated, and all other uses of these drugs were also brought under veterinary oversight. Although it is too early to fully evaluate compliance with the FDA’s guidelines in the United States, early signs indicate a reduction in the total sale of medically important antimicrobials in 2017 from the previous two years with a rebound in 2018 and 2019. The drop in the total sale of medically important antibiotics in 2017 was due to the implementation of GFI#213. Because their production use was discontinued in 2017, the quantity of these antibiotics used for therapeutic purposes has increased [19]. A similar scenario was also seen in Denmark following the ban of using antibiotics for growth promotion that led to a compensatory increase in the quantities of antibiotics used for therapeutic purposes [20]. It is important to note that FDA’s policy only affects medically important antibiotics, and the non-medically important antibiotics are still used for growth promotion purposes. Although these antimicrobial stewardships and their judicious uses are anticipated to reduce AMR associated with food animal production, its elimination is impossible. Therefore, improvement in animal manure management systems is equally important to reduce environmental contamination and eventually protect public health. Control of AMR, therefore, requires a “One Health” approach that involves multidisciplinary actions for an effective outcome.

The bulk of antibiotics in food animals are administered either in feed or water. For example, medically important antimicrobial drugs were overwhelmingly used in feed (65%) or in water (29%) in 2019 [19]. Although these two routes are also used for the treatment of individual animals, they are commonly used for the administration of antibiotics to many animals, usually a mixture of sick and healthy, for therapy, prophylaxis, or metaphylaxis. Even though this mass exposure to antimicrobials could lead to more AMR, it is a practical way to treat and prevent infections in poultry, swine, and beef cattle production systems where a group of animals are kept together in large numbers [4]. Nevertheless, oral administration of antibiotics exerts selection pressure on the gut microbiota favoring the selection of resistant strains and their antibiotic resistance, which can be excreted and spread into the environment through manure.

Intensive food animal production systems produce large quantities of manure, much of which is directly disposed of onto nearby crop land in the form of solid (manure) or liquid (slurry) waste, potentially increasing the risk of transfer of ARGs to bacteria in the environment [4,5]. Manure refers to animal excreta (feces and urine), litter (such as straw and feathers used for animal bedding), spilled feed and water, wash water, and other wastes [21]. Runoff from animal production facilities, manure storages such as stockpiles and ponds, and manure amended crop fields contain pathogens and ARGs, including tetracycline resistance (*tet*) genes, which can contaminate surface and ground water sources [22,23,24,25]. To reduce environmental pollution, farms use manure management technologies for disposal. These technologies reduce total manure volume, produce organic fertilizer, and some of them are tailored to generate biogas that can be used for on-farm energy needs in the form of biofuel [5]. However, current manure treatment systems have not been specifically designed to mitigate AMR [26]. Therefore, it is important to review their potential impacts in removing antimicrobial resistance genes from animal manure.

**Figure 1:**
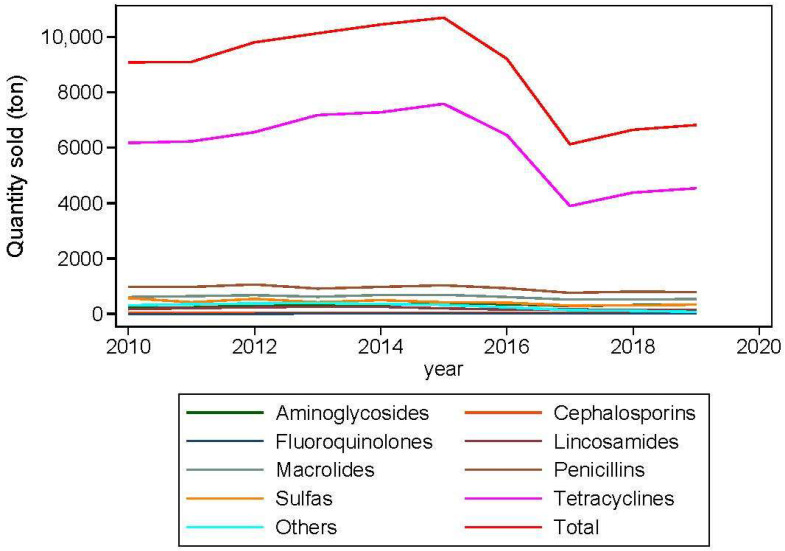
Medically important antimicrobial drugs approved for use in food-producing animals actively marketed in the United States between 2010–2019. Others: Amphenicols, Diaminopyrimidines, Fluoroquinolones (excluding 2013 through 2019), Polymyxins (excluding 2012 and 2013), and Streptogramins. Data were analyzed from FDA 2020 [19]. Guidance for Industry #213 [17] states that all antimicrobial drugs and their associated classes listed in Appendix A of FDA’s Guidance for Industry #152 [18] are considered “medically important” in human medical therapy.

We focused on tetracyclines since, as a class, they represent the largest volume of antibiotics sold for use in food-producing animals in the United States [19], Europe [27], and across the world [4]. As a class, tetracyclines represent the bulk of the total antibiotics sold in the United States between 2010–2019 (Figure 1). This widespread use of tetracyclines undoubtedly led to the widespread occurrence of tetracycline resistance [5]. In large time-series analyses, tetracycline resistance was the highest among historical *E. coli* isolates obtained from humans and food animals from 1950–2002 [28], *Salmonella* isolated from retail poultry regardless of antibiotic use claims of the product between 2008–2017 [29], and two major *Enterococcus* spp. isolated from retail meats between 2002–2014 [30]. Furthermore, tetracyclines are considered highly important class of antimicrobials according to the World Health Organization’s (WHO) categorization of medically important antimicrobials [31]. Three tetracycline resistance mechanisms have been described that involve the acquisition of genes that encode for (i) energy-dependent efflux of tetracyclines, (ii) proteins that protect bacterial ribosomes from the actions of tetracyclines, or (iii) enzymatically inactivate tetracycline [32]. Currently, 63 different tetracycline resistance genes belonging to efflux (36), ribosomal protection (13), enzymatic (13), and unknown (1) mechanisms have been identified (Table 1).

The *tet* genes are often associated with mobile genetic elements such as plasmids, transposons, and conjugative transposons, which are responsible for the horizontal transfer of ARGs. Horizontal transfer is another factor that explains the widespread occurrence of tetracycline resistance in a wide variety of bacterial species [32]. This review focuses on tetracycline resistance genes given their long-term and widespread use in animal agriculture. Furthermore, more than 75% of orally administered tetracycline is excreted unchanged or as an active metabolite in manure [33], making it a perfect candidate for the evaluation of interventions such as manure management technologies. Moreover, *tet* genes are the most abundant and frequently detected ARGs in animal manure [34,35].

In this review paper, we first briefly reviewed the operational practices of the three most common types of manure treatment, namely lagoon, anaerobic digestion, and composting [5], followed by reviewing their impact on *tet* genes in major food animal manure (cattle, swine, and poultry). We conducted a qualitative review with relevant keywords (manure, lagoon, anaerobic digestion, composting, tetracycline resistance gene) searched using PubMed and Google Scholar. Laboratory-scale studies were excluded, and we mainly focused on field studies. The current review is unique from previous reviews in a few important aspects. First, we reviewed manure management practices, both operationally and their impact on *tet* genes, in one place. Second, we focused on field-scale studies as they are more applicable on farms and represent real-life conditions. Third, we identified the weaknesses of published studies with respect to study design. Fourth, it focused on one antibiotic since the impact of animal manure treatments varies by antimicrobial type as some are more recalcitrant than others. Fifth, this review lays a foundation for scoping and systematic reviews, and meta-analyses on the impacts of manure management practices on antimicrobial-resistant bacteria and their associated resistance genes. Finally, this review identified important research gaps for future studies, such as the need for comprehensive risk analysis pertaining to the impact of animal manure treatments within the context of One Health in reducing the spread of antimicrobial-resistant bacteria.

## 2. Brief Description of Animal Manure Management Systems

Animal manure is treated to reduce waste volume with conversion to usable products such as nutrient-rich fertilizer, compost, or biogas [5]. The presence of antibiotics like tetracyclines in animal manure can create a continuous selection pressure for the development of the resistant bacterial population in these manure management systems with the potential for environmental spread [12,37,38]. Anaerobic digestion and lagoons are widely used methods for manure treatment [39] and provide a means of preparing animal manure for crop fertilization and gas production. Digestion reduces ARGs, including tetracycline resistance genes, which was a commonly held belief. Although this may be true of digesters operated under thermophilic conditions [40,41], other studies suggest otherwise, and *tet* genes may remain present at high concentrations [42]. Therefore, it is essential to evaluate animal manure management systems under field conditions regarding their effect on ARGs such as *tet*. This review summarizes literature regarding the impact of lagoons and anaerobic digestion, the two commonly used manure management systems both to produce biogas and biofertilizer, on *tet* genes. We also reviewed composting system for its potential use either as an independent method or as post lagoon and AD treatments to further remove ARGs.

### 2.1. Lagoons

A commonly used method of manure management on animal farms is flushing, usually in the form of a slurry, into an open-air basin known as a lagoon [43]. There, the slurry separates into a predominantly anaerobic liquid upper layer and a lower anaerobic sludge layer [44]. Although the upper layer typically receives some aeration, the degree of which will depend on local climatic conditions and wind fetch across the lagoon and the presence of wind barriers, lagoon treatment is mainly anaerobic in the absence of mechanical aeration [45,46]. A limited amount of anaerobic respiration may occur depending on the presence of alternative electron acceptors such as Fe^3+^, SO_4_^2−^, or NO_3_^−^, but these are usually quickly exhausted [47].

Lagoons can be single-celled or serially built multi-celled. Single-celled basins contain all the biological layers in one lagoon. On the other hand, in multi-celled basins, biological functions are partially split between the multiple cells [48]. Photosynthesizing microbes are found in the effluent storage layer. The treatment layer is composed of facultatively aerobic and anaerobic bacteria. The sludge storage layer houses the settled solids and supports anaerobic digestion. Herd size should be proportional to lagoon size and must provide storage for both the effluent and sludge. The dilution must be maintained as sludge and influent is added to the lagoon making sure not to reduce the volume of liquid beyond a minimum [48]. Effluent is removed from the upper layers of the cell basins and is often used as nutrients on crops without further treatment. This can spread antimicrobial-resistant bacteria and their associated resistance genes.

More advanced and specialized forms of lagoon manure management include anaerobic and aerobic lagoon systems. Anaerobic lagoons are covered lagoon digestion systems and are widely accepted in the United States for manure treatment [43]. Anaerobic treatment of manure helps to protect water quality by reducing much of the organic concentration of the material. Anaerobic lagoons also reduce the nitrogen content of the material through ammonia volatilization and effectively reduce manure odors if managed properly. For best performance, the optimum pH of an anaerobic lagoon is about 6.5 [49]. Anaerobic lagoons also produce methane that can be used for biogas production. They are more effective in warm climates where anaerobic microorganisms are sufficiently active year-round. Thus, their effectiveness can be affected by insufficient lagoon size, excessive daily manure loading, and cold temperature, leading to odor problems and poor anaerobic treatment and gas production [21]. Although not widely adopted, aerobic lagoons are used if minimizing odors is critical. They are designed based on the amount of biochemical oxygen demand (BOD) added per day and more surface area to allow for the oxygen entrainment that is necessary for aerobic bacteria. [49].

As the manure is biologically decomposed in the lagoons, undigested material, usually called sludge, is settled at the bottom layer, which should be removed when it exceeds the sludge storage capacity. Treated lagoon slurry is directly applied to soil as crop fertilizer without any further processing [50]. If improperly designed and managed, lagoon leakage around animal production facilities along with direct discharge can lead to surface and groundwater contamination [51,52]. The presence of antibiotics like tetracyclines in animal manure can create a continuous selection pressure for the development of the antibiotic-resistant bacterial population in these manure management systems with the potential for environmental spread. Microbial communities are segregated and play a unique part in the degradation process within the lagoon’s treatment and sludge storage layers [48]. Since, unlike anaerobic digesters, uncovered lagoons are open to the air, photosynthesizing bacteria may develop that act to reduce nitrogen and sulfur-containing compounds and help to eliminate odor in the effluent storage layer [48]. However, these bacteria have strong seasonal variation in their growth, and the environmental conditions under which they develop are not completely understood [53].

Feeding the lagoon with organic material ensures a continuous supply of nutrients and fresh microbes to sustain the biological digestion processes. Problems associated with liquid waste management include leakage, overflows, embankment failure, and odor emissions. Antibiotic contaminated manure in lagoons creates an ideal environment for the enrichment of antibiotic resistance genes by providing a continuous selection pressure on bacteria. Most lagoons are in direct contact with the ground and, although usually lined with sealants such as bentonite clay, exhibit some amount of leakage, and thus antibiotic-resistant bacteria and their ARGs can enter the underground watershed surrounding the environment [54,55]. Furthermore, since covered lagoons are passive systems, they are more effective in the warmer areas and seasons, and their efficiency drops when the ambient temperature falls below 20 °C [43].

### 2.2. Anaerobic Digestion

Anaerobic digestion is another common method of livestock manure management with the primary goal of producing methane for fuel [56]. The digested manure (i.e., the digestate) is used as fertilizer [56]. Like lagoon treatment, it is largely an anaerobic process with the provision made for capturing the produced biogas [57]. From the viewpoint of waste treatment, the most notable difference is that anaerobic digesters are often operated at a mesophilic temperature of 35 °C (range 25–40 °C) for 15–30 days, or thermophilic temperature of 55 °C (range 50–65 °C) to accelerate wastewater treatment and biogas production [39,43,58] rather than at ambient temperature. Although essentially anaerobic, laboratory-scale digester experiments have demonstrated that the addition of small amounts of air can result in the complete digestion of wastewater and greater biogas production. This process has been termed microaeration [59].

Anaerobic digestion, which also occurs in lagoons, uses microorganisms to break down organic matter in the absence of oxygen [43,57]. The primary goal of anaerobic digestion is biogas production through fermentation. In the case of lagoons, these are often covered to facilitate the capture of the biogas. Biogas consists of methane, carbon dioxide, and trace amounts of other gasses, including water vapor and hydrogen sulfide [60]. Biogas can be used to generate electricity or burned for heating and cooking [57]. In addition to biogas production, AD also reduces greenhouse gas emissions through methane capture and utilization, as well as odor management, pathogen control, soil amendment, and fertilization, and as animal bedding [61].

**Figure 2:**
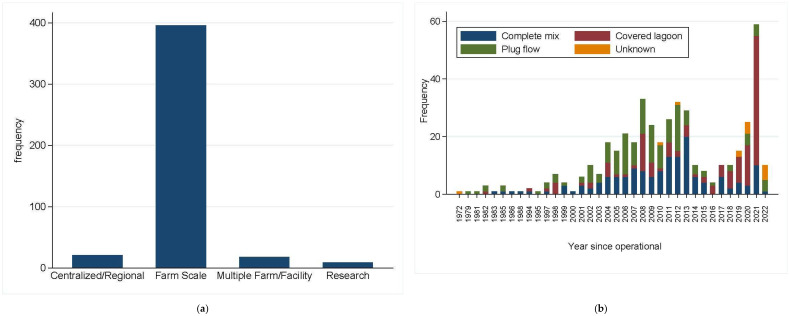

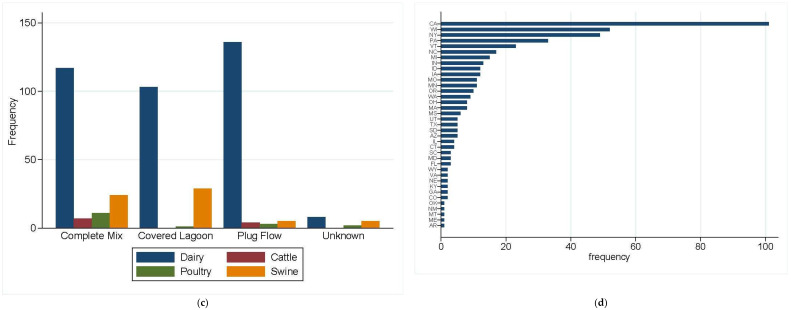
Anaerobic digestion systems registered in the United States for the treatment of animal manure. Frequency of anaerobic digestion facilities by (**a**) operation type; (**b**) year anaerobic digestion facility became operational; (**c**) animal production system (cattle refers to beef cattle); (**d**) State. Data were analyzed from the Agstar livestock AD database [62].

Anaerobic digestion of animal manure can be achieved through covered lagoons, plug flow digesters, and complete mix digesters [61]. According to the Agstar livestock AD database, from 1972–2022, there were 444 registered AD facilities in the United States [62] for treating food animal manure. Across the database, 317 (71.4%) AD facilities were operational; 38 (8.6%) of them were under construction, and the remaining 89 (20.1%) were closed. Over 89% of the AD systems were farm-scale (Figure 2a). The adoption of AD-based animal manure treatments steadily increased, with most AD facilities being constructed between 2001–2013, followed by a temporary decline before increasing again, peaking in 2021 (Figure 2b). Overall, covered lagoons, plug flow AD, and complete mix AD were found approximately at equal proportions each at one-third. However, the data shows a shift towards covered lagoons since 2018. Perhaps, this shift may be due to the covered lagoon’s low initial and operating costs as compared to above-ground anaerobic digesters. Across the entire dataset, the majority (82%, *n* = 364) of the AD operations were for use in dairy cattle, followed remotely by swine (14.2%), poultry (3.8%), and beef cattle (2.5%). We note there was overlap by animal species since there were 11 AD facilities with mixed animal species. Within the dairy operation, plug flow (37.4%) was the most frequently used, followed by complete mix (32.1%) and covered lagoon (28.3%), with the remaining 2.2% not specified. In swine, covered lagoon (46%; *n* = 63) was the most frequently used, followed by complete mix (38%) and plug flow (8%), with the remaining 8% not specified. In poultry, complete mix (64.7%, *n* = 17) was the most frequently used, followed by plug flow (17.7%) and covered lagoon (5.9%), with the remaining 11.8% not specified. In general, our analysis shows that AD systems are commonly used in dairy cattle production (Figure 2c), and the type of AD mostly used depends on the animal species, perhaps according to the consistency of the animal manure. The top five states that used the AD systems for the treatment of animal manure were CA, WI, NY, PA, and VT, respectively (Figure 2d). It should be noted that these are also the top dairy-producing states in the USA and that most AD facilities were initially built for use in the dairy cattle production system.

A properly constructed digester can reduce waste management, energy, and bedding costs and generate revenue for the farms by selling biogas. The two most common anaerobic digester types are plug flow and complete mix, constituting 70% of active farm digester types [63]. The plug flow digester, built as an elongated tank, can handle 11 to 13 percent solids suitable for scrapped manure; therefore, plug flow digesters are mainly used by the dairy industry because of the high density of the generated waste [60].

Complete mix digesters, built as an upright central tank with consistent agitation, are supplied through a continuous flow of animal manure. Complex digesters have a smaller footprint and are better suited for a more liquid waste than plug flow digesters but are slightly more expensive [60]. Some complex digesters are located partially or entirely below ground to take advantage of constant ground temperatures. The complex digester can only handle 3 to 10 percent solids. Biogas is piped off as it is produced, and often the liquid waste is piped into the bottom of the tank, with constant agitation to keep the waste material mixed. The waste that is piped in will move up the tank and through the microorganism-rich layers to speed up digestion. As waste is added to the tank, the effluent is removed to prevent overflow and to maintain a correct dilution.

Solid-liquid separation may be applied after AD in which undigested waste material from the anaerobic digestion processes is separated into solid and liquid wastes. Treated solids in the effluent are suitable for use as animal bedding, thus reducing a farmer’s production costs [64]. Furthermore, the material after digestion is less susceptible to bacterial growth because it has little to no organic material remaining. Liquid waste is either pumped back to a traditional storage facility (i.e., lagoon) or applied to crops as a fertilizer [56]. Essentially, all the nitrogen present in the animal waste is converted to ammonia by digestion, which is a critical ingredient in fertilizers [64].

### 2.3. Composting

Composting is a biological decomposition of organic matter in the presence of oxygen [56,65]. Although composting is a natural process, it can be enhanced and accelerated by mixing animal manure with bulking agents such as wood chips or straw for oxygen penetration and optimum microbial growth [39,65]. Composting converts organic waste into a stable organic product by converting nitrogen from unstable, volatile ammonia to a more stable form to be used as fertilizer [56,66]. Composting progresses through three temperature phases from the initial mesophilic (40 °C) phase to thermophilic (60 °C) as a result of bacterial action, followed by a final mesophilic (40 °C) phase to produce a cured product commonly known as compost [65,66]. Curing is the final stage of composting occurring after much of the readily metabolized biological material has been decomposed. It occurs at cooler temperatures than those in the thermophilic phase of composting. The curing process further reduces pathogens, promotes further decomposition of cellulose and lignin, and stabilizes composition. Curing may or may not involve insulation, depending on environmental conditions. The compost is generally safer than the raw animal manure and is used as organic fertilizer, reducing the bulk of animal manure, thus improving manure handling, reducing odor, eliminating vectors, and can destroy plant weeds and pathogens [65]. There are three basic methods of composting: windrow (turned piles), static pile, and in-vessel [66].

In the windrow method, the compost mix is arranged in long, narrow piles called windrows. The compost mixture is periodically turned, which exposes the compost mixture to the air to maintain aerobic conditions and to keep composting temperature from getting too high. The turning frequency varies from 2 to 10 days, depending on the type of mix, volume, and ambient air temperature, and can be reduced as the compost ages. The U.S. Food and Drug Administration (FDA) Food Safety Modernization Act (FSMA) [67] requires that turned composting should maintain aerobic conditions and a minimum temperature of 55 °C for 15 cumulative days with a minimum of five turnings followed by adequate curing. The static pile method involves mixing the compost material and then stacking the mix on perforated plastic pipe tubing through which air is drawn or forced. According to the US FSMA [67], static composting should maintain aerobic conditions at a minimum temperature of 55 °C for three consecutive days followed by adequate curing. The in-vessel method involves the mixing of manure or other organic waste with a bulking agent in a reactor, building, container, or vessel and may involve the addition of a controlled amount of air over a specific detention time. This method provides a high level of process control because moisture, aeration, and temperature can be maintained. The temperature-time requirement for in-vessel composting is similar to that of the static pile [39].

## 3. Effects of Animal Manure Treatments on the Removal of Tetracycline Resistance Genes

### 3.1. Impacts of Lagoons on Tetracycline Resistance Genes

A cross-sectional study [68] was conducted in swine farms by simultaneously sampling fresh manure and lagoon samples. Regular PCR was used for the detection of eight *tet* genes. Results indicated that the types and frequency of detection of the *tet* genes did not differ between fresh manure collected from the buildings and lagoon samples, suggesting the ineffectiveness of lagoon as animal manure treatment. In another cross-sectional study, *tet*(A) and *tet*(B), the only *tet* genes included in the qPCR array, were detected in 11 and 9 out of 12 pooled swine lagoon samples, respectively [22]. In a three-year longitudinal study that monitored swine waste lagoons at two production facilities, all targeted seven *tet* genes were consistently detected from all lagoon samples obtained from all the six sampling dates at variable concentrations [52]. These studies indicate that *tet* genes are readily detected in lagoons that are used to treat swine manure. We note that the studies did not properly evaluate the impact of lagoon treatment, which would require a control group or before and after types of study designs. Rather, the studies either collected a cross-section of both fresh manure and lagoon samples or periodically sampled the lagoons.

A comprehensive study [33] compared the effects of different lagoon treatment strategies on the concentrations of *tet*(O) and *tet*(W) in layer chicken, dairy and beef cattle, and swine farms. The authors observed that the levels of the *tet* genes were significantly lower in the chicken layer lagoons than the lagoons of all other animal operations. They also found significantly higher levels of the *tet* genes in the conventional dairy lagoons than the organic dairy. The authors also reported a significant seasonal effect: the *tet* concentrations were lowest in the summer compared to other seasons, suggesting that summer may be the optimal season for lagoon-treated manure (both effluent and biosolid) land application. Interestingly, concentrations of the *tet* genes tended to decrease as the manure effluents passed through multiple treatment lagoons arranged in series. Lagoon aeration did not have any significant effect on *tet* concentrations. The study concluded that although the use of sequential lagoons in series significantly reduced the *tet* concentrations, they did not completely remove them. In fact, the levels of the *tet* genes were 3–5 orders of magnitude above the background concentrations measured in pristine water sediments. Another study similarly reported the detection of *tet*(O) and *tet*(W) at concentrations of 8.3 and 8.9 log_10_ copies/mL of liquid samples obtained from a naturally aerated dairy lagoon [69], respectively.

In a study [70] that was conducted at three swine farms, the presence of 16 *tet* genes was investigated by PCR from community DNA obtained from feces/feed and lagoon samples. Their results indicated similar detection frequencies for six genes (*tet*(M) from one farm and a starter phase of a second farm), *tet*(O), *tet*(W), *tet*(G), *tet*(Q), and *tet*(X)) between fecal and lagoon samples; lower detection in the lagoon than feces/feed (*tet*(B), *tet*(L) and *tet*(D)); or higher in the lagoons (*tet*(A) and *tet*(M) from the finisher phase of one farm and one additional farm); and two genes (*tet*(K) and *tet*(T)) were not detected from any sample. The authors concluded that swine lagoons, while they can positively select for some *tet* genes, thereby increasing their proliferation, can also negatively select for some leading to their attenuation. To test the hypothesis that attenuation of a particular gene in lagoons may be the consequence of their initial low abundances in the upstream samples, the authors quantified the concentrations of selected genes using qPCR. Initial abundances of genes that were attenuated were similar to that of the genes whose concentrations were increased in the lagoon, suggesting that attenuation was real and was not the result of dilution of initially low abundant genes. Although the authors categorized samples as upstream (feces and feed) and downstream (lagoon) samples, there is no indication that the lagoon samples represented the treated samples of the upstream samples. Another swine study [71] also reported that three *tet* genes (*tet*(O), *tet*(Q), and *tet*(X)) were abundant in all lagoon samples examined by qPCR. All eight targeted *tet* genes (*tet*(O), *tet*(Q), *tet*(W), *tet*(M), *tet*B(P), *tet*(S), *tet*(T), and *otr*(A) were detected from the lagoon samples of two swine farms [72].

A study [73] conducted in three large commercial swine farms evaluated the effects of successive manure treatments on the level of *tet*(G). Farm A, a finishing farm that consisted of five barns, used a conventional lagoon (uncovered) into which manure slurry was directly discharged during the downtime of the biofilters. The authors reported that the conventional lagoon, which received untreated manure slurry, had a higher abundance of *tet*(G) than the feces samples. Farm B was a farrow-to-wean operation consisting of four gestation- and two farrowing- houses. Manure was flushed into a covered lagoon managed at ambient temperatures to produce biogas. Interestingly, the effluent from the covered lagoon was further treated by two successive nitrifying biofilters, thus enabling the evaluation of the impact of post-treatments. The study found that covered lagoon samples had a significantly lower abundance of *tet*(G) than the effluent from the swine houses that was fed into the digester. However, the difference was relatively small, with a 1.1 log reduction indicating that ARGs persisted despite the treatment effects. Following an increase in a storage pond after the first nitrification treatment, *tet*(G) gene decreased in the secondary biofilter by 3.4 logs, indicating the need for post-treatments after mesophilic anaerobic digestion in covered lagoons.

In a feedlot cattle study [74], the authors quantified three *tet* genes (*tet*(O), *tet*(W), and *tet*(Q)) from seven lagoons located at five feedlot cattle farms. The authors reported a strong association between the levels of the genes and tetracycline concentrations in the lagoon wastewater. In a similar study [75] that quantified six genes *tet*(O)*, tet*(Q)*, tet*(W)*, tet*(M), *tet*(B), and *tet*(L) from samples collected from eight lagoons from five cattle feedlots, a significant association was found between the levels of the *tet* genes and presumed qualitative levels of antibiotic use. Table 2 summarizes studies reviewed on lagoon treatment of animal manure.

### 3.2. Effect of Anaerobic Digestion on Tetracycline Resistance Genes

Factors known to affect the levels of ARGs, including *tet* genes, during AD of animal manure include pre-treatments such as advanced AD through a brief exposure of animal manure to high temperature (60–180 °C), solid-state (low liquid or garage digesters) AD, temperature, residence time, oxygenation (aerobic versus anaerobic digestion), and post-treatments [76]. Laboratory and pilot-scale digesters, although they provide the best evidence due to effective control of AD parameters, they may not provide real-life results [77]. Because of economic and logistical considerations, full-scale ADs at commercial food animal production farms often operate as continuous or semi-continuous reactions with shorter residence times [77]. Therefore, in this review, emphasis was given to full-scale anaerobic digesters to summarize the effect of AD on *tet* genes. Lab- or pilot-scale anaerobic digesters were referenced sparingly.

A study [73] was conducted in a finishing swine farm to quantify the effect of high-solid mesophilic anaerobic digestion of separated solids for biogas production on *tet*(G). As in the covered lagoon anaerobic digestion of swine farm described above, the high-solids anaerobic digestion had a similar abundance of the *tet*(G) gene as post digestion separated solids and higher *tet*(G) gene than the raw, undigested manure. Higher abundance in the separated solids and the digestate, when compared to the original manure in the concentration of the *tet*(G) gene, may be due to its adsorption to solid matrices [38]. Results also indicated that *tet*(G) persisted during mesophilic anaerobic digestion of swine manure, also perhaps through adsorption to solids. The authors [73] concluded that AD at mesophilic temperatures has little to no effect on removing ARGs, including tetracycline resistance genes, as also previously reported in lab-scale studies [12,38]. Thermophilic AD was shown to be more effective than mesophilic AD in reducing *tet* genes in human wastewater treatment [78]. However, since thermophilic AD is more expensive, it is not widely used on animal farms, and its future adoption requires economic incentives. Furthermore, thermophilic temperatures (≥55 °C) may impair the performance of the digesters with respect to biogas production [41], a primary incentive for the use of AD systems.

The impact of an advanced AD facility at a large dairy cow farm in which manure was mixed with food-grade organic wastes on *tet*(O) and *tet*(W) was evaluated [69]. Pretreated by pasteurization and hydrolysis, the mixtures were subjected to mesophilic AD for 22 days. The log gene copies of the two *tet* genes measured did not differ among raw manure, pasteurized manure, and digested manure samples. This study further supports the ineffectiveness of mesophilic digestion to remove ARGs such as *tet* genes from animal manure.

A study [41] evaluated the impact of aerobic digestion, conventional aeration of wastewater with externally applied air, and anaerobic digestion of human wastewater solids in lab-scale digesters operated under four temperatures on the concentrations of five *tet* genes. Significant reductions were observed in the anaerobic digestion with increased removal rates and efficiencies as a function of temperature. The authors concluded that high temperature (≥37 °C) AD could be an effective technology for eliminating ARGs such as *tet* genes from wastewater solids. On the other hand, aerobic digestion with much shorter residence times than the AD had a less substantial effect on the levels of the *tet* genes measured. The authors also pointed out that with longer residence times (>15 days), thermophilic aerobic digestion may achieve similar impacts as thermophilic AD. In general, the authors concluded that thermophilic AD could be very effective at reducing the quantities of *tet* genes in wastewater solids. However, the high cost of operating full-scale thermophilic AD operated at longer residence times makes it impractical for the treatment of animal manure [41]. In addition, higher temperatures for longer residence times can compromise methane production for biogas which is the main goal of AD [76]. Therefore, the temperature-residence time combination should be optimized against maximizing biogas production and operational cost. One suggestion to achieve thermophilic conditions at no extra cost is by heating up anaerobic digesters using solar energy, especially during the summer season [76].

Solid-state AD (20–40% solid concentration) has higher efficiency for lignocellulose digestion, smaller digester capacity, and lower energy than the conventional liquid (<15% solids) AD [56]. Compared with liquid AD, solid-state AD reduced the abundances of some of the ARGs considered in the study [79], including *tet* genes (*tet*(C), *tet*(G), *tet*(W), and *tet*(X)). While this study was limited to *tet* genes, another study that evaluated on-farm anaerobic digestion of dairy manure reported that AD, while it abates some ARGs, it increased others such as the macrolide resistance genes *erm*(B) and *erm*(F) [80] or had no effect on other resistance genes [77].

The most comprehensive longitudinal study [77] to date evaluated the impact of full-scale AD on ARG concentrations. The study was conducted at seven dairy farms that used either plug flow or complete mix digesters. Raw and digested manure samples were collected biweekly over nine months. The authors quantified concentrations of *tet*(A) and *tet*(W). Results indicated that the mean concentration of *tet*(A) decreased during AD, consistent with a previous study [38] of AD of swine manure. On the other hand, the authors [77] reported that the mean concentration of *tet*(W) remained unchanged, as similarly reported in the pilot-scale AD of swine manure [12]. Although the authors [77] reported seasonal variations in the mean concentrations of the *tet* genes, the performance of the AD did not vary by season with respect to its effect on the *tet* concentrations. The authors, once again, concluded that AD is limited as a tool to mitigate ARGs in animal manure and that multiple on-farm interventions, such as judicious use of antibiotics, are needed to effectively remove ARGs before animal manure is released into the environment.

There are conflicting reports on the effect of season on *tet* gene concentrations during AD of animal manure suggesting the need for more field-scale studies in different animal species. For example, in one study [77] on AD of dairy manure, concentrations of *tet*(A) and *tet*(W) were significantly highest in the summer. On the other hand, in another study [69], concentrations of *tet*(O) and *tet*(W) genes did not significantly differ between spring and winter months in the effluent samples collected after anaerobic digestion of dairy manure.

Variation due to animal species has been noted on the effectiveness of AD on animal manure in reducing *tet* genes [12,77]. For example, while the mean concentration of *tet*(A) increased during AD of beef cattle and swine manure, it did not change in the poultry litter. On the other hand, *tet*(W) increased during AD of poultry litter with no changes in the cattle and swine manure [12]. However, studies comparing the effect of AD by animal species are scarce, possibly indicating the widespread use of AD in the dairy cattle industry more so than in the swine and poultry production [61] (see also Figure 2).

Many dairy farms in the US use solid-liquid separation (SLS). It is reported that SLS promotes the sorption and partitioning of ARGs to separated solids [26]. Tetracyclines are known to partition with the separated solids [81]. Hence AD of separated solids may efficiently reduce ARGs in animal manure. Solid-state AD, also referred to as garage AD, is typically performed in batch mode. Furthermore, the digestate from AD can be processed using SLS separating the digested manure into solid and liquid fractions. While the solid fraction can be used as a bedding material or composted, the liquid fraction can be recycled for barn flushing or used for irrigation [56]. Pre-treatments such as thermal pre-treatments had shown some promise. Pasteurization of dairy manure at 67 °C, while significantly reducing concentrations of sulphonamide resistance genes, did not significantly affect concentrations of *tet* genes despite a significant reduction in tetracycline concentration [69]. Table 3 summarizes the main conclusions of selected studies that evaluated the effect of anaerobic digestion of animal manure on tetracycline resistance genes.

### 3.3. Impact of Composting on the Removal of tet Genes from Animal Manure

Studies [56,69,76] suggest the need for post-treatments such as composting of anaerobically digested animal manure to reduce the concentrations of ARGs. Sequential use of composting following the recovery of useable energy from raw manure after AD seems an acceptable and cost-effective technology. The critical temperature-time regimens of composting are stipulated by federal regulations to ensure the reduction in pathogens [39]. Furthermore, composting is a low-cost technology suitable for both small and large livestock operations with increased demand for organic farming. Therefore, it is essential to evaluate the impact of composting on the removal of ARGs, including *tet* genes.

A field study [82] evaluated the impact of a 10-week composting of poultry manure spiked with the quinolone antibiotics ciprofloxacin and enrofloxacin, as well as doxycycline on ARGs. Results indicated that out of eight *tet* genes evaluated, the concentrations of seven genes (*tet*(A), *tet*(B), *tet*(K), *tet*(M), *tet*(Q), *tet*(S), and *tet*(W)) were significantly reduced (up to 98.9–99.99% reduction) at the end of composting. However, the concentration of *tet*(Y) was significantly increased. The concentration of doxycycline also decreased significantly by composting. The authors concluded that composting of poultry manure is an effective method to reduce (if not completely remove) the concentrations of most ARGs, including the *tet* genes. This selective effect of composting on genes, even those in the same antimicrobial class, may be due to microbial succession and the potential for increased mobility of ARGs that may occur during composting [13].

A comprehensive study [13] was conducted to evaluate the effect of turned and static composting of manure obtained from dairy and beef cattle with or without standard antibiotic treatments. Composting was done for 42 days using small-scale composters using aerated static composting or daily turned small-scale composters. Periodically obtained samples were analyzed for resistome composition using shotgun metagenomic sequencing and qPCR for *tet*(W) quantification. A significant reduction in the relative abundance of total ARGs across all compost conditions was observed at the end of composting. At the individual antimicrobial class level, the abundance of *tet* genes showed a striking decrease during composting. Similarly, both absolute and relative abundances of *tet*(W) significantly decreased across all compost conditions from day 0 to day 42.

To compare the effectiveness of stockpiling and composting in reducing ARGs in beef cattle manure, Staley et al. [24] conducted field experiments using bulking agents. The experiments were conducted during the winter-spring cycle and summer-fall cycle. Although the concentrations of the *tet* genes studied (*tet*(O) and *tet*(Q)) did not significantly change during the winter-spring cycle, composting resulted in significant reductions in their concentrations during the summer-fall cycle. This suggests the seasonal effect on the effectiveness of composting. During the cold months, significant fluctuations in the temperature profile in the composting piles affect composting performance. The authors also pointed out that bulking agents can be a direct source of preexisting ARGs or support the growth of ARB present in the manure.

#### Composting as Post Anaerobic Digestion Treatment

Few studies have evaluated the use of composting, as a post-animal manure digestate treatment, on *tet* genes. In a completely randomized study [83], the authors compared the effects of composting solid poultry litter digestate or solid digestate with co-composting materials (undigested poultry litter, food processing waste, or maize silage) on the abundances of four *tet* genes (*tet*(K), *tet*(M), *tet*(O), and *tet*(S)). Materials were composted for 90 days. The initial concentrations, in log_10_/g, of the *tet* genes in the solid digestate were 7.5 (*tet*(K)), 9.0 (*tet*(M)), 7.0 (*tet*(O)), and 5.0 (*tet*(S)). *tet*(M) was the most abundant ARG in the solid digestate, whereas *tet*(O) and *tet*(S) were the least abundant, as similarly reported in the solid digestate of poultry litter, swine, and cattle manure [12]. The reported concentrations of the *tet* genes in the poultry litter digestate once again suggest the incomplete removal of ARGs by anaerobic digestion and the need for post-digestion treatments before land application. The co-composting materials also contained the *tet* genes with the initial concentration ranging from 3.6 log_10_ *tet*(O) in the food processing waste to 11.3 log_10_ *tet*(M) in the poultry litter. Poultry litter had the highest initial concentrations of the *tet* genes, followed by solid poultry litter digestate, while maize silage and food processing waste had the lowest concentrations.

The authors [83] reported that >80% of all ARGs were removed after 90 days of composting, but removals from co-composting were lower. The greatest reductions were observed during the thermophilic phase suggesting the importance of composting temperature in removing the ARGs, perhaps through inactivation of bacteria carrying them. Enrichment after the initial reductions during the thermophilic phase occurred during the mesophilic phase for most of the *tet* genes. For example, *tet*(K) concentration significantly increased during the mesophilic phase, although it decreased during the maturity phase. On the other hand, enrichment of *tet*(S) rarely occurred, and *tet*(O) had the greatest reduction in all composted materials. Interestingly, the greatest reductions in the abundances of the *tet* genes were observed in the solid poultry litter digestate. In the final composts, poultry litter solid digestate compost had the lowest abundance of the *tet* genes, while poultry litter solid digestate co-composted with fresh poultry litter had the highest abundance. It can be concluded that co-composting materials, especially poultry litter, may promote or can be the source of ARGs during composting of anaerobic digestate. It was noted that *tet*(K) and *tet*(M) were enriched when food processing waste and maize silage were co-composted with solid digestate. This indicates that using these materials as bulking agents for post-digestate composting is not satisfactory, and perhaps it may increase the abundances of tetracycline resistance genes rather than abating them. Table 4 summarizes the results of composting studies reviewed for their effect on tetracycline resistance genes. 

## 4. Conclusions

Our review revealed that *tet* genes were commonly detected in abundant concentrations in the lagoons of swine, dairy cattle, and feedlot cattle farms. Lagoon studies were mostly conducted in swine production facilities. Correlations between the *tet* concentrations and the level of tetracyclines measured in the lagoon samples were variable. This shows the need for more research on the upstream use of antibiotics to examine its impact on the downstream performance of animal manure management practices. Our review clearly indicates that field lagoon systems commonly operated at ambient temperatures do not affect the removal of tetracycline resistance genes.

In general, thermophilic AD (operating temperature 55–60 °C) systems were found to be more effective than mesophilic AD (operating temperature 35–37 °C) both under full-scale field studies [78] and in lab-scale studies in removing ARGs, including *tet* genes from human waste solids [41]. Thermophilic AD, solid-state AD, and advanced AD systems are more effective than mesophilic and conventional AD systems in reducing the concentrations of ARGs in animal manure.

Field studies report inconsistent results regarding the effectiveness of composting in removing ARGs from animal manure. While some studies have reported a consistent decrease in ARG abundance during composting, others noted inconsistent effects, with some ARGs decreasing while others persisted or even increased. This inconsistency may be attributed to variability in manure characteristics, type of bulking agent, ambient temperature, pile operation (such as turning frequency), and sampling strategy of the compost pile [24]. Several studies investigated changes in *tet* gene content during composting with inconsistent conclusions.

## 5. Research Gaps and Future Directions

None of the reviewed studies evaluated the impact of lagoons as animal manure treatment on the concentrations of *tet* genes under controlled experimental design. An appropriate design would be to follow batches of fresh manure during lagooning (i.e., a longitudinal design). A continuous sampling scheme would be a better sampling strategy since, in most cases, manure is flushed into lagoons periodically, and that mixing will occur in the lagoon between flushes. None of the studies did this. Rather, they collected lagoon samples at different time points and measured the outcomes, either presence/absence or concentrations. In other words, the studies were not conducted as before and after design. The hydraulic retention time for manure in the lagoons was not reported among the studies. Only one study [70] reported 2–3 months retention time in their field study.

Research is needed to elucidate the mechanisms by which the quantities of ARGs decline, sustain, or possibly increase during the digestion of animal manure [41]. Proper control groups or well-designed before and after trials with large sample sizes are required to effectively evaluate manure treatments. Most of the studies used qPCR for the quantification. Although qPCR targets the genotypes, rather than phenotypes, which can be horizontally transferred within the animal manure treatment systems, it does not provide information on whether the genes are functional or if they were from dead or live bacteria [41]. Further research is needed to determine the role of horizontal gene transfer and composition of bacterial communities carrying the ARGs during AD [56]. Metagenomic studies will provide a deeper and broader understanding of both the resistomes and microbiomes impacted by a particular treatment method. Molecular techniques that can link the resistomes with the microbiomes are essential to target AMR spread through animal manure.

Some studies suggest post AD treatments such as composting can facilitate further removal of ARGs [56]. However, a comparison of AD and composting on the abundance of *tet* genes in animal manure is required within the same experiment under field conditions. No studies were found in the literature that concurrently compared AD and composting. Studies that sequentially compared composting as a post AD treatment are lacking.

In general, manure treatment systems are not designed to mitigate AMR, although some have more capacity than others. Well-managed aerobic composting with >60 °C is more effective than static piles; thermophilic anaerobic digesters operating under steady-state are more effective than mesophilic or irregularly operated digesters or anaerobic lagoons [26]. Although thermophilic digesters are more effective, both in methane production and ARGs removal, than the mesophilic conditions, they require more expensive technology and an increased level of technical operation and monitoring [39]. The effects of animal manure treatments on the abundance of *tet* genes are variable due to three main factors: (1) multiplication or death of the bacteria carrying ARGs, depending on the conditions of the manure treatments; (2) presence of antibiotics and heavy metals in the manure that can exert selection pressure on ARG carrying bacteria and; (3) the possibility of the horizontal spread of ARGs among bacteria [35]. Future studies are needed to harness the values of animal manure management as waste disposal and resource utilization as potential mitigation strategies to reduce the spread of antimicrobial resistance.

Quantitative risk analysis is required within a “One Health” concept to quantify the risk associated with the use of antibiotics both in animals and humans with respect to environmental and public health. There are few quantitative risk analyses performed on the use of antibiotics in animals and their risk to human health as reviewed by McEwen [84]. However, comprehensive quantitative risk analysis along the animal production-environment-public health continuum is lacking. Moreover, comparative quantitative risk analysis to quantify the risk of environmental (soil and water) contamination from human wastewater is required. This is very important because a previous study [22] showed that treated human waste discharged from municipal wastewater treatment plants had a higher prevalence and diversity of antimicrobial resistance genes than beef cattle ponds or swine waste lagoons. Quantitative risk analysis would evaluate the measures to be taken to reduce the burden of antimicrobial resistance, such as good antimicrobial stewardship to reduce overall upstream antibiotic use and the downstream wastewater management practices.

## Figures and Tables

**Table 1:** Tetracycline resistance genes identified by their mechanism of resistance [36].

Efflux (36)	Ribosomal Protection (13)	Enzymatic Degradation (13)	Mosaic Ribosomal Protection (11)	Unknown
*tet*(A), *tet*(B), *tet*(C), *tet*(D), *tet*(E), *tet*(59) *tet*(G), *tet*(H), *tet*(J), *tet*(V), *tet*(Y) *tet*(Z), *tet*(30), *tet*(31), *tet*(33), *tet*(57) *tet*(35) *tet*(39), *tet*(41) *tet*(K), *tet*(L), *tet*(38), *tet*(45), *tet*(58), *tet*(63) *tetA*(P), *tet*(40) *otr*(B), *otr*(C) *tcr3* *tet*(42) *tet*(43) *tet*AB(46) *tet*AB(60) *tet*(62) *tet*(64)	*tet*(M), *tet*(O), *tet*(S), *tet*(W), *tet*(32) *tet*(Q), *tet*(T), *tet*(36), *tet*(61) *otr*(A), *tetB*(P), *tet* *tet*(44)	*tet*(X) *tet*(37) *tet*(34) *tet*(47), *tet*(48), *tet*(49), *tet*(50) *tet*(51), *tet*(52), *tet*(53), *tet*(54) *tet*(55), *tet*(56)	*tet*(O/32/O), *tet*(O/W/32/O), *tet*(O/32/O) *tet*(O/W/32/O/W/O), *tet*(W/32/O), *tet*(O/W) *tet*(W/32/O/W/O), *tet*(O/W/O), *tet*(O/W/32/O) *tet*(S/M), *tet*(W/N/W)	*tet*(U)

**Table 2:** Tetracycline resistance genes targeted and detected/quantified from animal waste lagoons.

Reference	*tet* Genes Targeted	Animal Operation	Conclusions
[68]	M, O, Q, W, A, C, H, Z	Swine	No substantial difference. M, O, Q, and W were detected at 100% from all building and lagoon samples tested at all farms. *tet*(A) was detected only from one building sample at one farm. *tet*(C) was detected from building samples at two farms, and lagoon samples at one farm; lagoon samples showed a 20% lower prevalence than building samples for *tet*(C). H and Z were detected from all tested samples; H showed a 20% lower prevalence in one farm, while Z showed a 30% higher prevalence.
[52]	M, O, Q, W, C, H, Z	Swine	All seven genes were detected at 100% prevalence from all lagoon samples and six sampling dates at the two swine farms. Concentrations fluctuated during the three-year monitoring period, with an average concentration of 1.42 × 10^4^ copies per 10^6^ 16S rRNA copies.
[22]	A, B	Swine	Occurred at 92% *(tet*(A)) and 75% (*tet*(B)) prevalence from pooled samples
[33]	O, W	Chicken layer, dairy cattle, beef cattle, swine	The *tet* genes tended to decrease in concentration as the animal waste effluents passed through multiple treatment lagoons, ranging from 0–1 log reduction depending on animal spp. However, no complete removal.
[69]	O, W	Dairy cattle	8.3 and 8.9 log_10_ copies/mL, respectively
[70]	16 genes	Swine	Three genes (G, M, X) persisted and amplified ~100–1000 fold; two genes (B, L) were attenuated in the lagoons. Others were similar between feces and lagoon samples.
[75]	M, O, Q, W, B, L	Cattle feedlot	2.8 × 10^6^ copies/mL high use lagoons; 7.3 × 10^5^ copies/mL in mixed use lagoons; 5.1 × 10^3^ copies/mL in no-use lagoons
[73]	G	Swine	1.1 log reduction in the covered lagoon; post-treatment resulted in 3.4 log reduction
[71]	O, Q, X	Feedlot cattle, swine	The average relative abundance of ARGs ranged from 5.5 × 10^−6^ to 6.3 × 10^−1^ copies per 16S rRNA gene.
[72]	W, O, Q, M, S, T, B, *otr*(A)	Swine	All genes were detected from the lagoon samples
[74]	O, W, Q	Feedlot cattle	Concentration ranged from 2.8–4.3 logs/50 µL

**Table 3:** Fate of tetracycline resistance genes during anaerobic digestion of animal manure.

Genes	Change in Abundance	Type of Digestion	Manure Type	Reference
*tet*(G)	No change	Mesophilic	Swine	[73]
*tet*(O)	No change	Advanced mesophilic after pre-digestion pasteurization	Dairy cattle	[69]
*tet*(W)				
*tet*(A)	Decreased by 0.7 log_10_	Mesophilic	Dairy cattle	[77]
*tet*(W)	No change			

**Table 4:** Effect of composting of animal manure on tetracycline resistance genes.

Gene	Manure Type	Bulking Agent	Compost Type	Composting Duration	Change	Reference
*tet*(A), *tet*(B), *tet*(K), *tet*(M), *tet*(Q), *tet*(S), *tet*(W)	Poultry litter	Barley straw	Windrow, turned once/week	10 weeks	Decreased (by 2.5 logs on average)	[82]
*tet*(Y)					Increased (by 0.7 logs)	
*tet*(W)	Dairy manure	Alfalfa hay, pine bark mulch, and sawdust	Static composting	42 days	Decreased (1–2 log reduction)	[13]
	Feedlot cattle		Turned composting, turned daily			
*tet*(O), *tet*(Q)	Feedlot cattle	Ground corn stalks	Turned after days 49 and 112 for the winter-spring cycle; no turning for the summer-fall cycle	140 days	Up to 2 log reduction	[24]
*tet*(K), *tet*(M), *tet*(O) and *tet*(S)	Solid poultry litter digestate	Alone or with co- composting materials	Turned weekly, biweekly, and trice weekly at each composting stage	90 days	>80% reduction	[83]

## Data Availability

Not applicable.
